# Surgical treatment of a complex craniocervical malformation combined with posterior cranial fossa teratoma: a case report and literature review

**DOI:** 10.1186/s41016-020-00230-0

**Published:** 2021-01-18

**Authors:** Jiang Liu, Rui He, Chao Wang

**Affiliations:** 1 Department of Neurosurgery, The First Affiliated Hospital of USTC, Division of Life Sciences and Medicine, University of Science and Technology of China, Hefei, 230036 Anhui People’s Republic of China; 2 Department of Orthopedics, The First Affiliated Hospital of USTC, Division of Life Sciences and Medicine, University of Science and Technology of China, Hefei, 230036 Anhui People’s Republic of China; 3 Department of Orthopedics, Peking University Third Hospital, Beijing, 100191 China

**Keywords:** Atlantoaxial dislocation, Skull traction, Anterior transoral release, Teratoma

## Abstract

**Background:**

Basilar invagination (BI) with atlantoaxial dislocation (AAD) is not uncommon in patients with scoliosis, Klippel-Feil syndrome (KFS), and other bone deformities. Cases with combinations of the abovementioned dislocations and deformities with posterior cranial fossa teratoma are rare in the clinic and difficult to handle.

**Case presentation:**

This case presents a 34-year-old woman diagnosed with atlantoaxial dislocation and posterior cranial fossa mass. After two surgeries, the posterior cranial teratoma was completely removed with satisfactory atlantoaxial reduction. The postoperative 1-year follow-up examination showed that the bone graft fusion was successful, without remaining significant dysfunction.

**Conclusions:**

The surgical risk of irreducible atlantoaxial dislocation combined with posterior cranial fossa tumor is huge. Thus, it needs to be fully preoperatively evaluated and managed carefully in accordance with sound surgical principles.

## Background

Complex craniocervical malformations are not uncommon in clinical practice, but reports of combined posterior cranial fossa tumors are rare. Therefore, the ideal surgical choice for such patients remains controversial. Here, we report the case of a patient who underwent two surgeries with satisfactory results.

## Case presentation

**Fig. 1:**
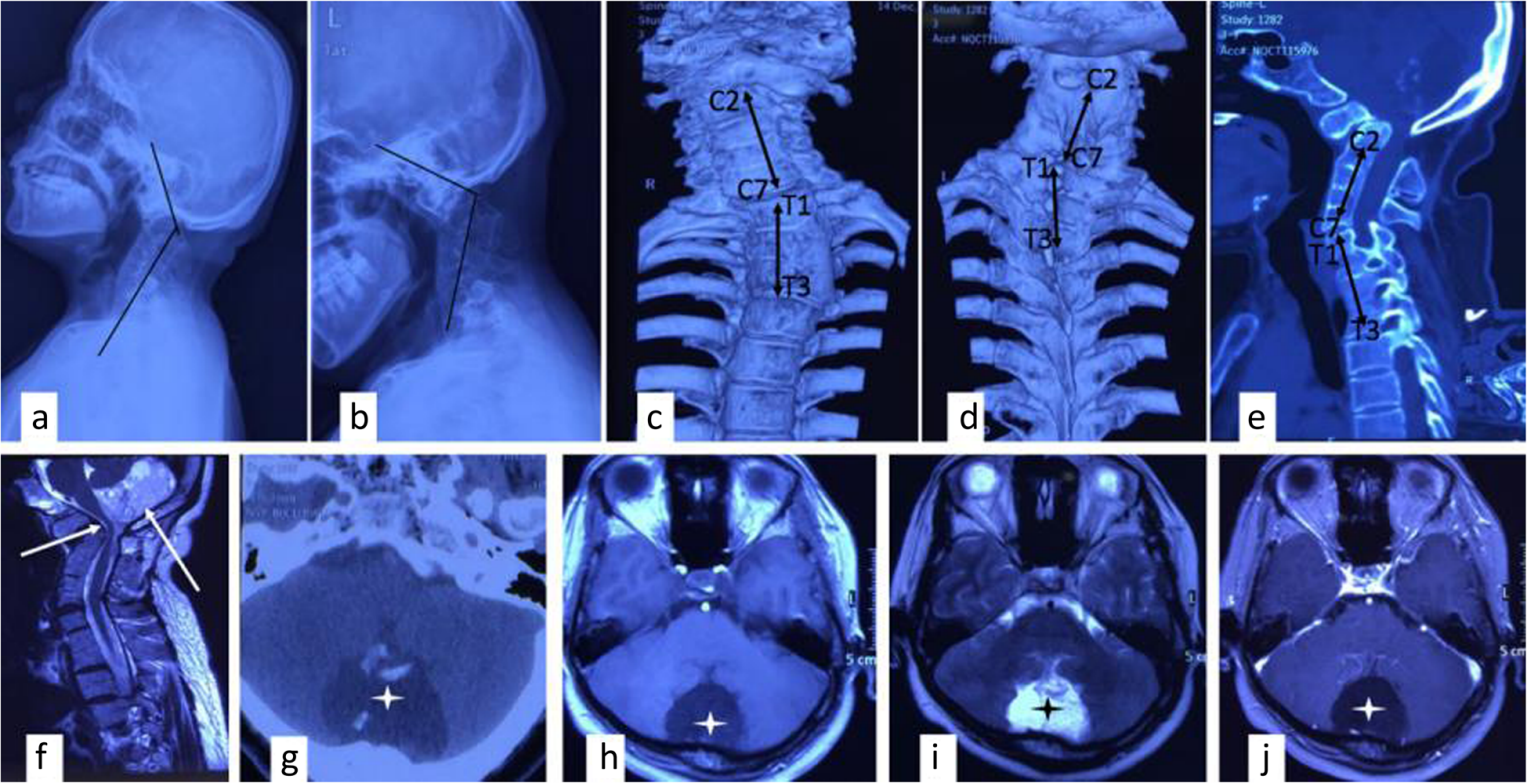
Preoperative imagings. **a**, **b** Flexion and extension of cervical spine demonstrated AAD with KFS. **c**–**f** Reconstructive CT and MRI showed severe KFS with scoliosis and observable compression of the cervical-medullary. **g**–**j** CT and MRI scan showed the lesion in posterior cranial fossa

A 34-year-old female patient was admitted to the hospital with a complaint of numbness and weakness in the right upper limb for 4 months. No previous medical history was available. Physical examination showed that her neck was short, with low posterior hairline, cranial nerve sign (-), right upper extremity with motor power of 4/5, Romberg’s sign (+), and Babinski’s sign (+). Imaging examination, including plain X-ray, computerized tomography (CT) scanning, and magnetic resonance imaging (MRI), revealed severe atlantoaxial dislocation with multi-segmental vertebrae fusion of the cervical and thoracic spine. The compression on the ventral side of the foramen magnum was obvious. Additionally, the mass in the posterior cranial fossa was considered a congenital tumor based on the characteristics of the image presented in Fig. 1.

**Fig. 2:**
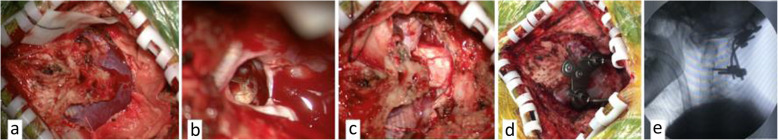
Intraoperative photos of the first operation. **a**–**c** Excision of the posterior fossa lesion through suboccipital craniectomy, the lesion was pearly white with hair inside. **d**, **e** The dura mater was tightly repaired followed by occipitocervical fixation, and intraoperative X-ray showed partial reduction of atlantoaxial dislocation

Resection of the posterior cranial fossa tumor and occipitocervical fixation and fusion were simultaneously performed from a single posterior approach. Since the SUMMIT occipitocervical internal fixation system (DePuy Spine, Raynham, MA, USA) was adopted, the bone at the midline of the occipital was retained to facilitate the placement of the occipital plate. A near-total excision of the lesion was done from the small window craniectomy on both sides of the posterior cranial fossa. The tumor consisted of hair, several fragments of bone tissue, and mature adipose. Upon removal of most of the tumor, the dura mater was tightly repaired and sutured, followed by occipitocervical fixation and fusion. During the operation, we found that the patient had osteoporosis, and thus the bilateral C2 pedicle was easy to split when the screw was implanted; no reduction existed by applying posterior thumb pressure, by anteriorly compressing the C2 spinous process [1]. Finally, we had little choice but to insert a lateral mass and laminar screw. Then, intraoperative X-ray showed partial reduction of AAD was obtained (Fig. 2).

**Fig. 3:**
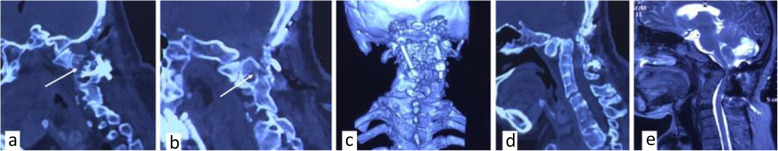
CT and MRI scan after the first operation. **a**, **b** CT sagittal reconstruction image revealed the bilateral C2 pedicle were split (white arrow). **c** Three-dimensional reconstructive CT showed the bone window and the occipital plate. **d**, **e** The tumor resection was relatively satisfactory, but the atlantoaxial dislocation became even worse, indicated loss of the reduction compared the intraoperative C-arm radiograph

Unfortunately, the numbness and weakness of the limbs were not relieved after the operation, and the patient had difficulty walking because of her unsteady gait. Histopathology of the posterior cranial fossa lesion indicated of “mature teratoma”. Postoperative imaging showed that the tumor resection was relatively satisfactory, but the atlantoaxial dislocation became even worse. The compression of the brainstem and the upper cervical cord was increased, which was consistent with the clinical manifestations (Fig. 3).

**Fig. 4:**
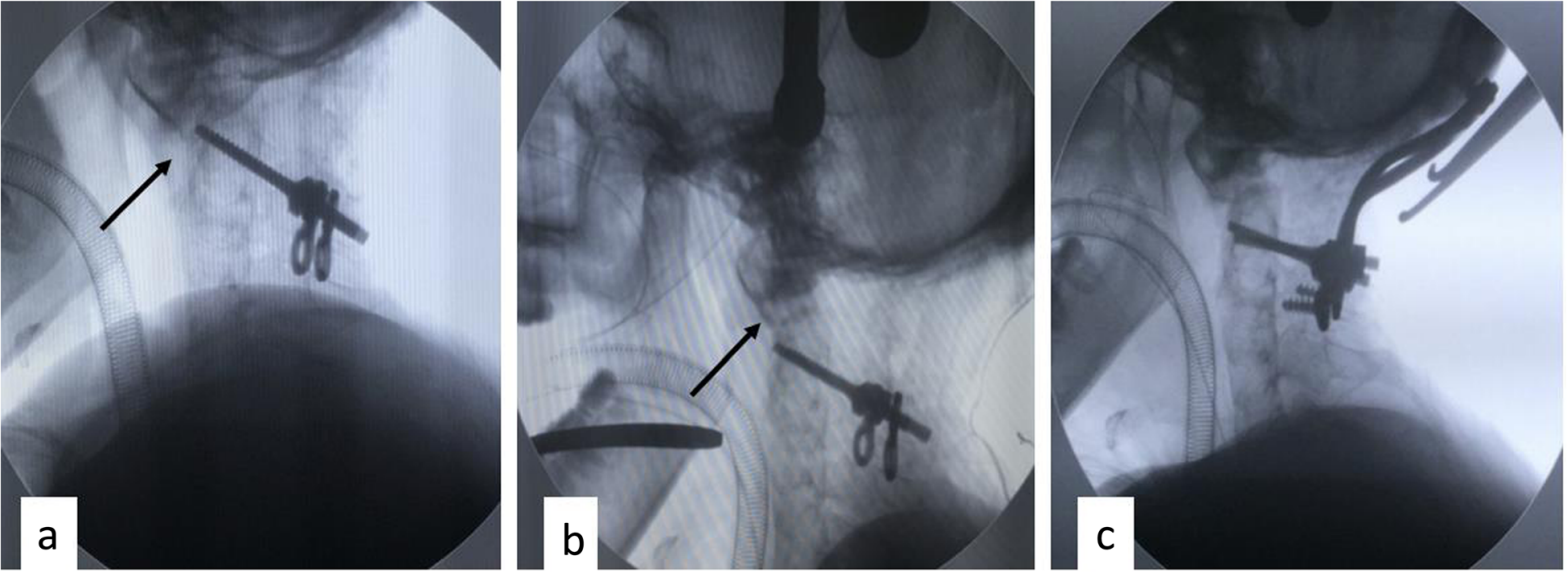
Intraoperative C-arm radiograph in the revision surgery. **a** C2 pedicle screws were inserted. **b** After anterior transoral release, the tip of the odontoid was clearly brought down. **c** The posterior occipitocervix fixation was performed

Therefore, a revision operation was performed 1 month later. Under general anesthesia, continuous skull traction (10 Kg) was maintained during the procedure. First, we extracted the previously inserted internal fixation system from the posterior approach. Next, 3.5-mm-diameter uniaxial C2 pedicle screws were inserted gingerly due to the proximal existence of a high-riding vertebral artery (HRVA). The position of the C2 pedicle screws was suitable, but the AAD reduction was unacceptable as assessed on the lateral intraoperative radiograph. Then, we performed anterior transoral release with carefully maintained traction during the transition from the prone to supine position. As the soft tissues and ligaments were released, a reduction of the atlantoaxial joint was progressively achieved until the tip of the odontoid was clearly brought down. The procedure was confirmed using C-arm imaging. Finally, posterior occipitocervical fixation fusion was performed immediately by a combination of C_2_ pedicle screws and an occipitocervical plate system while turning the body position again. To increase the screw control force, the bilateral C3 lateral mass was additionally set concurrently [2] (Fig. 4).

**Fig. 5:**
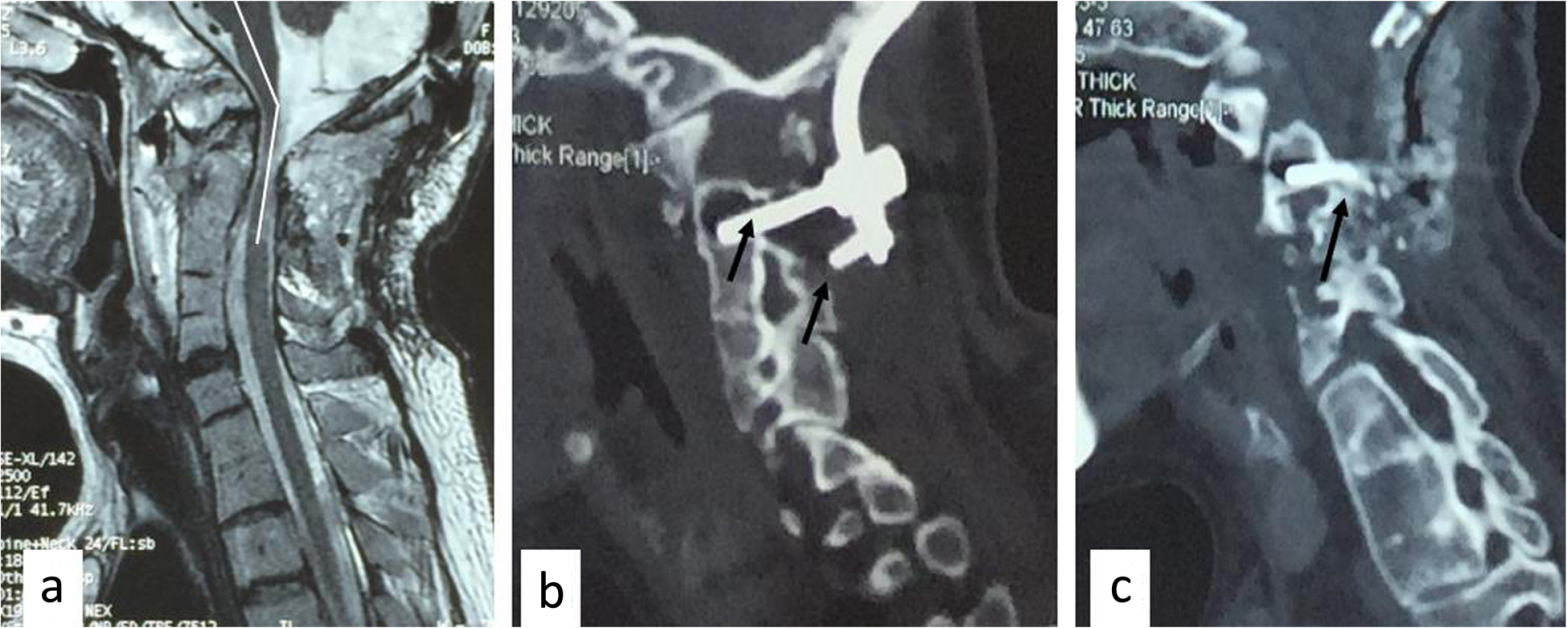
CT and MRI scan after revision surgery. **a** The compression of the ventral side of the brainstem and the upper cervical cord was significantly relieved, and the cervicomedullary angle returned to normal. **b**, **c** CT sagittal reconstruction image showed screws loosening and pull-out

After the second operation, the patient’s symptoms improved significantly. Postoperative MRI indicated that the compression of medulla oblongata and the upper cervical cord was significantly relieved, and the cervicomedullary angle returned to normal. However, obvious loosening and pull-out of bilateral C2 pedicle screws and C3 lateral mass screws were found on the CT sagittal reconstruction image (Fig. 5). Hence, Halo-Vest immobilization was immediately performed. Later, the patient experienced fever and headache due to intracranial infection confirmed by lumbar puncture. She was cured by continued lumbar cerebrospinal fluid drainage and was safely discharged from the hospital 10 days postoperatively.

**Fig. 6:**
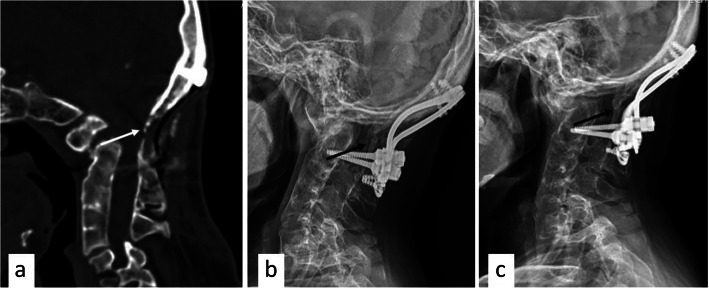
Images at follow-up. **a** Four months after revision surgery, CT showed the bone graft between the occipital and axis had fusion signs (white arrow). **b**, **c** Half a year and 1 year after revision surgery, X-ray confirmed a solid fusion was achieved and the internal fixation was stable (black arrow)

The patient was closely followed-up. Four months after the operation, CT showed fusion signs in the bone graft between the occipital bone and the axis had. The Halo-Vest fixation was removed half a year after the operation, when the lateral radiograph of the cervical spine showed a bone bridge had been formed in the occipitocervical junction. One year after operation, X-ray confirmed that a solid fusion was achieved and the internal fixation was stable (Fig. 6b, c). Further, the 2-year follow-up examination revealed a successful clinical outcome as she was able to return to her normal life as a housewife.

## Discussion

To the best of our knowledge, cases of craniocervical complex malformation combined posterior cranial fossa tumor have been rarely reported so far [3–5]. Details are summarized in Table 1. Therefore, the surgical experience for the management of such patients is not sufficiently rich. Upon reviewing the case, we established that the compression of the ventral side of the brainstem and the upper cervical cord caused by AAD was the main cause of the disease. Thus, the optimal treatment should be aimed at decompression by restoring the normal sequence of the upper cervical spine, along with surgical excision of the mass. However, this surgery is challenging, mainly due to the long operation time and complicated operating procedures, including the use of a microsurgery technique combined with an internal fixation technique. It is noteworthy that the opening of the dura mater and transoral procedure increase the chance of infection and cerebrospinal fluid leakage [6], and occipital craniectomy is unavoidable for excision of the tumor. Meanwhile, the area available for implant fixation and the occipitocervical fusion is influenced.

**Table 1:** Patients diagnosed with “posterior fossa lesion & CVJ malformation” and undergoing surgery reported in the literature [3–5]

Patient	Age (years)	Sex	CVJ malformation	Histopathology of the posterior fossa lesion	Occipitocervical fusion	Follow-up
1	25	M	BI, reducible AAD, C0-C1 and C2-3 fusion	Dermoid cyst	Occiput C2-C3 (using inverted U-shaped, contoured rod)	Good effect
2	23	M	BI, reducible AAD, C0-C1 fusion	Dermoid cyst	Occiput C2-C3 (using a contoured rod)	Good effect
3	18	F	BI, C2-3 fusion, irreducible AAD	Epidermoid cyst	Excision of the odontoid process via the transoral route + occipitocervical (occipital C2–C3) fusion	Good effect
4	12	F	Reducible AAD; C0-C1 fusion; C2-5 fusion	Dermoid cyst	Occipitoaxial fusion (using multiholed plates)	Good effect
5	20	M	Platybasia, BI, C0-C1 fusion, C2-3fusion	Dermoid cyst	Excision of the posterior fossa lesion by Poppen’s approach followed by transoral odontoidectomy and posterior fusion 2 months later.	Good effect

According to the surgical classification of AAD of Wang et al. [7], this case was categorized as irreducible AAD based on the reducibility established by skull traction under general anesthesia. Therefore, the treatment strategy for irreducible AAD is anterior transoral release of soft tissues and the ligaments around the odontoid process, followed by a posterior internal fixation and fusion [7–9]. The patient’s revision surgery also confirmed that this combined anterior-posterior approach was effective to achieve decompression. Screw loosening was considered to be related to severe osteoporosis in such patients [10] and stress concentration of internal fixation because of multi-segment cervical fusion. Therefore, preoperative radiography should be carefully evaluated, and postoperative external fixation measures should be taken forcefully without delay.

Choosing multistage surgery may be feasible in such cases due to the impossibility of completion of the operation by a posterior-only approach in only one stage. For these patients, the transoral atlantoaxial reduction plate (TARP) surgery [11, 12] is perhaps a reasonable option to initially perform a direct anterior fixation and achieve stabilization for resetting the atlantoaxial joint and providing decompression via the transoral route. Tumors in the posterior cranial fossa can be temporarily observed and treated by the posterior approach in a second surgery.

Certainly, even if TARP operation is performed, the risk is still considerable. Because the extensive fusion of the cervical spine will inevitably lead to a very large stress on the screw, there is a considerable possibility that external fixation assistance will be required after surgery. In addition, in a previous study, the patient’s occipital condyle dysplasia, wedge-shaped atlas lateral mass, atlantoaxial lateral sagittal joints appeared almost vertical [13]. Therefore, short-segment internal fixation including occipital condyle screws [14] and atlantoaxial fixation [15] are difficult to implement for screw placement. We consider that it is necessary to extend the fixed segment to achieve strong occipitocervical fixation and fusion in patients with KFS-type I [16].

The current standard occipitocervical fusion involved internal rigid fixation by polyaxial screws in the cervical spine, contoured rods, and occipital plate. In the first operation on this patient, we routinely chose the SUMMIT occipitocervical internal fixation system. Apparently, the Y-shaped titanium plate fixed to the occipital midline bone definitely affected the exposure of the tumors and the bone graft fusion. If we were to treat a similar patient again, it would be a better choice to apply an occipitocervical fixation system using an occipitocervical plate-rod system [15] or cervical pedicle screws and an occipitocervical plate [17] fixed to the lateral occipital bone. Meanwhile, we suggest that the posterior fossa craniectomy should be as small as possible to facilitate an occipitocervical fusion. Excision of the posterior fossa lesion with endoscopic assistance helps in preserving the suboccipital bone in these patients. It is also beneficial for removing the tumor as much as possible and looking around the corners [3, 5].

For AAD and KFS patients, long pedicle screws for C2 fixation carry the risk of damaging the vertebral arteries with HRVA [18]. Even so, to reduce the revision rate, we do not recommend the use of pars screws and translaminar screws for their poor biomechanical performance [19]. Goel et al. [18] proposed a surgical option for C2 screw fixation in cases with HRVA by exposing and mobilizing the vertebral artery. Preoperative computed tomography angiography (CTA) images and reconstruction views facilitate the evaluation of the suitability of screw insertion, which should decrease vertebral artery injuries during screw insertion [20]. In addition, it is necessary to extend the fixed segment to maintain the stability of the internal fixation.

## Conclusion

Here, we report a case of a patient with complex craniocervical malformation accompanied by posterior cranial fossa teratoma, for which there is currently no standard surgical method. Full evaluation is needed before surgery as well as careful management in accordance with sound surgical principles. In patients with irreducible AAD and KFS-type I, an appropriate occipitocervical fixation method should be selected on the basis of anterior release. It is a wise choice to extend the fixed segment. In view of the existence of posterior fossa lesions, while correcting the craniocervical deformity, it is necessary to consider the position and size of the posterior fossa craniectomy and the influence on the bone graft fusion.

## Data Availability

Not applicable.

## Abbreviations

AAD: Atlantoaxial dislocation
BI: Basilar invagination
CT: Computed tomography
CTA: Computed tomography angiography
HRVA: High-riding vertebral artery
KFS: Klippel-feil syndrome
MRI: Magnetic resonance imaging
TARP: Transoral atlantoaxial reduction plate
